# MicroRNA-132, Delivered by Mesenchymal Stem Cell-Derived Exosomes, Promote Angiogenesis in Myocardial Infarction

**DOI:** 10.1155/2018/3290372

**Published:** 2018-09-09

**Authors:** Teng Ma, Yueqiu Chen, Yihuan Chen, Qingyou Meng, Jiacheng Sun, Lianbo Shao, Yunsheng Yu, Haoyue Huang, Yanqiu Hu, Ziying Yang, Junjie Yang, Zhenya Shen

**Affiliations:** Department of Cardiovascular Surgery of the First Affiliated Hospital & Institute for Cardiovascular Science, Soochow University, Suzhou, China

## Abstract

**Background:**

To cure ischemic diseases, angiogenesis needs to be improved by various strategies in ischemic area. Considering that microRNA-132 (miR-132) regulates endothelial cell behavior during angiogenesis and the safe and efficacious delivery of microRNAs *in vivo* is rarely achieved, an ideal vehicle for miR-132 delivery could bring the promise for ischemic diseases. As a natural carrier of biological molecules, exosomes are more and more developed as an ideal vehicle for miRNA transfer. Meanwhile, mesenchymal stem cells could release large amounts of exosomes. Thus, this study aimed to investigate whether MSC-derived exosomes can be used for miR-132 delivery in the treatment of myocardial ischemia.

**Methods:**

MSC-derived exosomes were electroporated with miR-132 mimics and inhibitors. After electroporation, miR-132 exosomes were labelled with DiI and added to HUVECs. Internalization of DiI-labelled exosomes was examined by fluorescent microscopy. Expression levels of miR-132 in exosomes and HUVECs were quantified by real-time PCR. The mRNA levels of miR-132 target gene RASA1 in HUVECs were quantified by real-time PCR. Luciferase reporter assay was performed to examine the targeting relationship between miR-132 and RASA1. The effects of miR-132 exosomes on the angiogenic ability of endothelial cells were evaluated by tube formation assay. Matrigel plug assay and myocardial infarction model were used to determine whether miR-132 exosomes can promote angiogenesis *in vivo*.

**Results:**

miR-132 mimics were effectively electroporated and highly detected in MSC-derived exosomes. The expression level of miR-132 was high in HUVECs preincubated with miR-132 mimic-electroporated exosomes and low in HUVECs preincubated with miR-132 inhibitor-electroporated exosomes. The expression level of RASA1, miR-132 target gene, was reversely correlated with miR-132 expression in HUVECs pretreated with exosomes. Luciferase reporter assay further confirmed that RASA1 was a direct target of miR-132. Exosomes loaded with miR-132, as a vehicle for miRNA transfer, significantly increased tube formation of endothelial cells. Moreover, subcutaneous injection of HUVECs pretreated with miR-132 exosomes in nude mice significantly increased their angiogenesis capacity *in vivo*. In addition, transplantation of miR-132 exosomes in the ischemic hearts of mice markedly enhanced the neovascularization in the peri-infarct zone and preserved heart functions.

**Conclusions:**

The findings suggest that the export of miR-132 via MSC-derived exosomes represents a novel strategy to enhance angiogenesis in ischemic diseases.

## 1. Introduction

In acute ischemic diseases such as myocardial ischemia, blood flow to the heart is impaired. Vessels need to be regenerated to rescue the ischemic cascade. Neoangiogenesis can be improved by activating endogenous progenitor cells, supplying exogenous stem cells and/or therapeutic molecules such as angiogenic mRNA or microRNAs [1, 2].

MicroRNAs, a class of small noncoding RNAs (containing about 18–22 nucleotides), regulate gene expression by direct binding to the 3′-untranslated region (3′-UTR) of their target mRNAs and inducing their translational inhibition and/or degradation. MicroRNAs are recognized to participate in biological development, cell differentiation, apoptosis, and many other physiological and pathological processes [3]. Recently, multiple lines of evidence indicate that miR-132 regulate many processes in endothelial cells including angiogenic responses [1, 4, 5]. In 2010, Anand et al. demonstrated that upregulation of miR-132 positively controls pathological angiogenesis in response to vascular endothelial growth factor A (VEGF-A) by suppressing p120RasGap (RASA1) [4]. Recently, a study conducted by Katare et al. reported pericyte progenitor cells constitutively expressed and secreted miR-132 and promoted endothelial angiogenesis via modulation of methyl-CpG-binding protein 2 (MeCP2) [1]. Moreover, the potent proangiogenic effect of miR-132 has been confirmed in a mouse hind limb ischemia model. The study suggested that miR-132 may exert their proangiogenic effect by enhancing the Ras-mitogen-activated protein kinases (MAPK) signaling pathway through direct inhibition of RASA1 and Spred1 [5].

Exosomes are nanosized extracellular vesicles (30–100 nm in diameter) and are positive for CD9, CD63, and CD81. As a type of membrane vesicle, exosomes now have been recognized as a vehicle to facilitate intercellular communication and modulate the function of recipient cells though delivery of proteins, RNA, and other molecular constituents [6]. Exosomes have many remarkable attributes, such as stability, biocompatibility, and low immunogenicity, that delivery vehicles should have. The wide distribution of exosomes in the blood, urine, bronchoalveolar lavage fluid, breast milk, synovial fluid, pleural effusions, and ascites demonstrated that exosomes are well tolerated in biological fluids [7]. Another highly desired attribute of delivery vehicles is the ability to home to target location. Accumulating evidence suggests that depending on their cell source, surface antigen, and contents, exosomes could target specific cell types [8, 9]. These attributes provide a rationale for the applications of exosomes as therapeutic delivery vehicles in a wide spectrum of diseases such as cardiovascular disease, kidney injury, immune disease, neurological diseases, and cancer [9, 10–13].

The contents of exosomes are cell type specific and vary from different pathological conditions [14, 15]. Appropriate cell types need to be considered to obtain optimal and plentiful exosomes. Recently, mesenchymal stem cells (MSCs) are reported to be capable of secreting a large amount of functional exosomes. Studies have also demonstrated that MSC-derived exosomes have a significant proangiogenic function in myocardial infarction (MI) and hind limb ischemia model [10, 16].

In this study, we proposed that miR-132, delivered by MSC-derived exosomes, could exert the angiogenic effect in myocardial infarction. We investigated the angiogenic effect of miR-132-electroporated exosomes derived from MSCs *in vitro* and *in vivo*, as well as the underlying mechanisms. We treated HUVECs with miR-132 exosomes and found that miR-132 was upregulated in recipient cells, while the target gene RASA1 was downregulated in HUVECs. miR-132 exosomes promoted angiogenesis of HUVECs both *in vitro* and *in vivo*. In addition, transplantation of miR-132 exosomes in the ischemic hearts of mice markedly enhanced the neovascularization in the peri-infarct zone and preserved heart functions. Our study represents a potential strategy for revascularization and has important implications for new therapeutic approaches to ischemic diseases.

## 2. Materials and Methods

### 2.1. Animals

The mice were purchased from the Laboratory Animal Center of Nanjing University (Nanjing, China). The animals were housed under specific pathogen-free conditions, with 12-hour light/dark cycles and free access to food and water. The animal experiment was approved by the Ethic Committee of Soochow University. All efforts were made to minimize animal suffering.

### 2.2. Cell Culture

Bone marrow-derived mesenchymal stem cells (BMSCs) were isolated based on a previously reported procedure [17]; bone marrow cells were flushed from the bone cavity of femurs and tibias using 1 ml syringe with low-glucose Dulbecco's modified eagle medium (DMEM). All bone marrow cells were passed through a 70 *μ*m cell strainer. The obtained bone marrow cells were seeded onto a culture dish and incubated at 37°C in a humidified atmosphere containing 5% CO_2_, with C57BL/6 mouse mesenchymal stem cell growth medium (Cyagen, Guangdong, China). The phenotype profile of BMSCs (P4–P6) was identified by flow cytometry, using antibodies against mouse CD31, CD44, and CD105 and Sca-1. Human umbilical venous endothelial cells (HUVECs; Cell Bank of Chinese Academy of Sciences, Shanghai, China) were cultured in EGM2 supplemented with 5% fetal bovine serum according to manufacturer's instructions. All experiments were performed before passage 7.

### 2.3. Isolation and Purification of MSC-Derived Exosomes

MSCs were cultured in DMEM/F12 supplemented with 10% exosome-free FBS. After 48 h, exosomes were isolated from BMSC supernatant as previously described [10]. Briefly, the supernatant was obtained and centrifuged at 200 ×g for 30 min at 4°C to remove cellular debris. Afterwards, the supernatants were mixed with total exosome isolation reagent (Invitrogen, USA) overnight at 4°C. After centrifuging at 10,000 ×g for 1 h, the pellet was then carefully resuspended in 200 *μ*l of PBS and used immediately or stored at −80°C. To analyze these exosomes, the characteristic surface marker proteins of exosomes were analyzed by Western blot and the exosome morphologies were observed with a transmission electron microscope (TEM) (JEOL JEM-1230) as described previously in detail.

### 2.4. Loading miR-132 into Exosomes

Resuspended exosomes were diluted in the Gene Pulser electroporation buffer (Bio-Rad Laboratories, CA) in 1 : 1 ratio. 1 *μ*mol of mouse miR-132 mimic (Ambion, NY) or inhibitor was added to 200 *μ*l of exosome sample. The mixtures were transferred into cold 0.2 cm electroporation cuvettes and electroporated at 150 V/100 *μ*F capacitance using a Gene Pulser II system (Bio-Rad Laboratories, CA) as described previously [18]. After removing the free-floating miRNA mimic, exosomes were reisolated using ultracentrifugation. The final pellet (exosome) was resuspended in PBS and stored at −80°C.

### 2.5. Exosome Labelling and Internalization

Exosomes (250 *μ*g) were labelled with 1 *μ*M of DiI lipophilic dye (Invitrogen). After incubating at 37°C for 30 min, excess dye was removed by washing with PBS, and labelled exosomes were reisolated by ultracentrifugation (described above). Recipient HUVECs (3 × 10^5^) were incubated with DiI-labelled exosomes (10 *μ*g) for 2 h, fixed in 4% paraformaldehyde (PFA) for 10 min at room temperature, washed with PBS for three times, incubated with DAPI (1 : 500, Invitrogen) for 5 minutes at room temperature, and subjected to confocal microscopy using a Zeiss LSM 780 confocal microscope with 100x magnification (*n* = 3).

### 2.6. Tube-Like Structure Formation Assay

2 × 10^4^/well HUVECs (three replicates per group) were seeded on top of Matrigel (BD Biosciences) in a 96-well plate and treated with the blank exosomes, miR-132 mimic electroexosomes, or miR-132 inhibitor electroexosomes, respectively. After incubation at 37°C for 6 h, tube formation was observed by an inverted microscope (Leica DMI6000B, Germany), and the cumulative tube length of the network structure was quantified (4x magnification) using ImageJ software.

### 2.7. Matrigel Plug Angiogenesis Assay

Matrigel plug angiogenesis assays were performed as previously described [19]; 2.5 × 10^5^ HUVECs were treated with 30 *μ*g of exosomes, or vehicle control (DMEM), premixed with Matrigel (1 mg/ml, BD Biosciences) and DMEM, and injected subcutaneously into SCID male mice (6-week-old, *n* = 6) in both inguinal regions. After 14 days, the animals were sacrificed using overdose of anesthetic. Plugs were excised and performed to the subsequent immunofluorescence assay.

### 2.8. Acute MI Model and Assessment of Heart Functions

An acute myocardial infarction (AMI) was generated in mice as described previously [20]. Briefly, C57BL/6J mice (female, ~20 g) were anesthetized with ketamine (80 mg/kg, IP) and mechanically ventilated. The left anterior descending artery (LAD) was ligated with a 6-0 suture, and the animals were divided into four groups: saline control, miR-132, Exo-null, and Exo-132. After LAD ligation, each mouse received an intramyocardial injection of PBS, miR-132, normal exosome, or miR-132-electroporated exosome, respectively. A total of 20 *μ*l saline containing PBS, miR-132, or exosomes (600 *μ*g) was transplanted by myocardial injection near the ligation site in the free wall of the left ventricle.

Cardiac function was determined by performing echocardiography on days 3, 7, and 28 after MI, using the Vevo 2100 system (VisualSonics Inc., Toronto, ON, Canada) with an 80 MHz probe. The left ventricular parameters were recorded from two-dimensional images using the M-mode interrogation in the short-axis view. Finally, the mice were sacrificed to harvest the heart tissue for immunohistochemical analysis.

### 2.9. Immunohistochemical Analysis

Immunohistochemistry staining was performed to detect vessel density of Matrigel plug and heart tissue as described previously [21]. The fresh tissue samples were fixed in 4% paraformaldehyde (PFA) and then embedded in OCT and cut into 6 *μ*m thick slices. After blocking with 3% bovine serum albumin (BSA) for 30 min, the sections were subsequently incubated overnight at 4°C with the primary antibody against CD31. Secondary antibody goat anti-mouse Alexa 594 (1 : 500; Life Technologies) was used for detection. The nuclei were counterstained with 4,6-diamidino-2-phenylindole (DAPI). Images were observed by using a fluorescence microscope (Olympus).

### 2.10. Dual-Luciferase Reporter Assay

To elucidate whether RASA1 was a target gene of miR-132, TargetScan (http://targetscan.org) was used to predict miRNA molecules that may regulate RASA1, and miR-132 was identified as a potential regulator of RASA1. Wild-type (WT) and mutant seed regions of miR-132 in the 3′-UTR of RASA1 gene were cloned into pMIR-REPORT luciferase reporter plasmids (Invitrogen, USA). Plasmids with WT or mutant 3′-UTR DNA sequences were cotransfected with miR-132 mimic (100 nM; Sangon Biotech Co. Ltd., Shanghai, China) or negative control mimics into HEK293T cells (ATCC, Manassas, VA, USA). After cultivation at 37°C for 24 hours, cells were assayed using the dual-luciferase assay system (Promega, Madison, USA) according to the manufacturer's instructions. All assays were repeated at least three times.

### 2.11. Quantitative RT-PCR Assay

Total RNA was isolated from exosomes or HUVECs using TRIzol reagent (Invitrogen, USA) as described previously [22], and reverse transcription was performed using the microRNA reverse transcription system (GenePharma, Shanghai, China) or the PrimeScript RT reagent kit (TAKARA, Japan). The expression level of miR-132 was analyzed by SYBR Green assay following the manufacturer's instruction, using U6 as control. For RASA1, quantitative RT-PCR (Q-PCR) was performed using SYBR PCR master mix in the ABI Step One-Plus Detection system (Applied Biosystems, USA) according to the manufacturer's instructions. The primers used for RASA1 are as follows: sense, 5′-TTATGATGGGAGGCCGCTATT-3′, and antisense: 5′-CTGCATTGGTACAGGTTCCTT-3′. GAPDH was used as an internal control. The 2−ΔΔCT method was employed to determine the relative mRNA expression. Each assay was performed in triplicate.

### 2.12. Western Blot Analysis

Western blotting was performed to quantify specific protein expression levels in BMSCs and BMSC-derived exosomes. Samples were lysed with RIPA buffer containing protease inhibitor cocktail (Roche, USA), and the protein concentration was determined by BCA assay (Roche, USA). Equal quantities of protein were loaded and run on 10% SDS-PAGE gels and then transferred to polyvinylidene difluoride (PVDF) membranes. Each membrane was blocked in 5% BSA and subsequently incubated overnight at 4°C with anti-CD9 and anti-CD63, respectively. After washing, the membranes were incubated with peroxidase-conjugated goat anti-mouse secondary antibody (Invitrogen, USA). Image analysis and blot quantification were performed with Image Quant LAS 4000 mini biomolecular imager (GE Healthcare, Uppsala, Sweden).

### 2.13. Statistical Analysis

All data of *in vitro* experiments were obtained from at least three independent experiments. In the *in vivo* study, more than 6 samples were used in each group. The results were presented as means ± SD unless otherwise indicated and were analyzed using GraphPad Prism 5 software. Statistical analyses were performed using a two-tailed Student *t*-test or one-way ANOVA with post hoc tests to determine significant differences between the groups. *P* < 0.05 was considered statistically significant.

## 3. Results

### 3.1. Characterization of BMSCs and BMSC-Derived Exosomes

**Figure 1:**
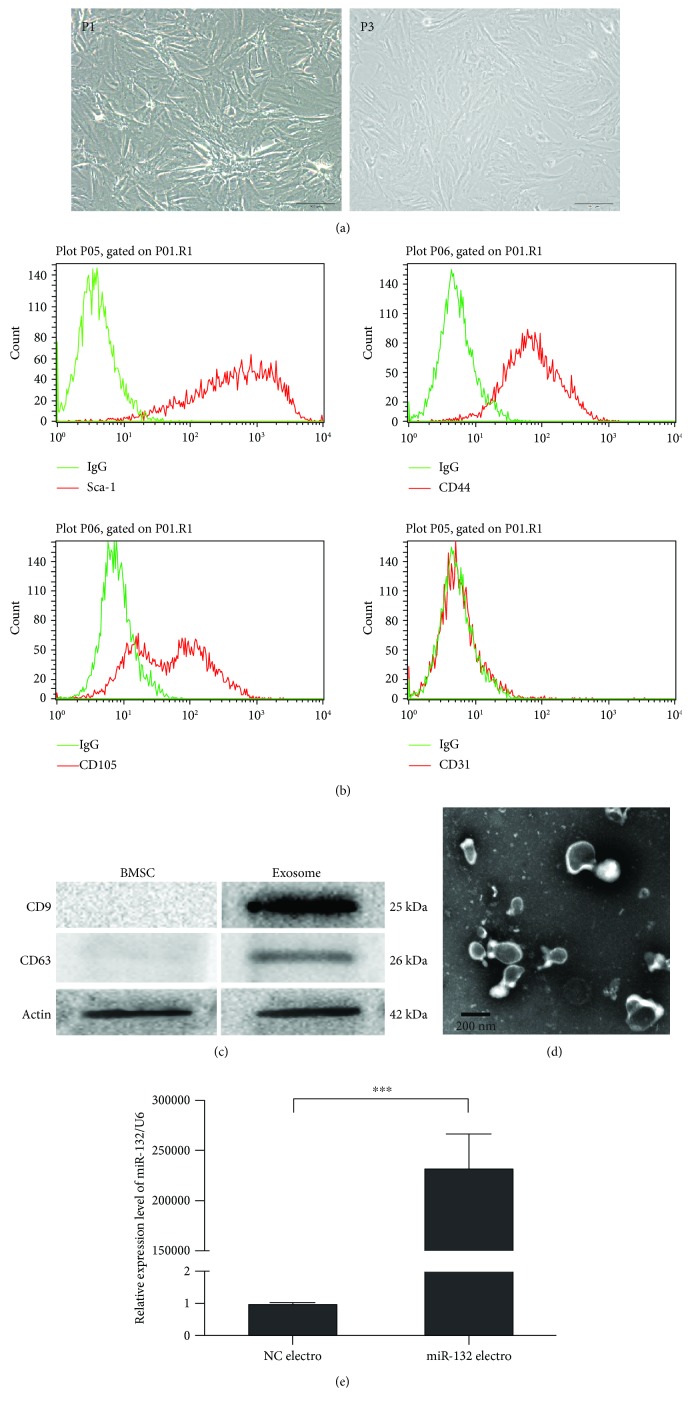
Characterization of BMSCs and BMSC-derived exosomes. (a) Morphology of MSCs (P1, P3) observed under an inverted fluorescence microscope. Scale bar: 100 *μ*m. (b) Phenotypic analysis of cell surface antigens of MSCs by flow cytometry (*n* = 3). (c) Surface marker proteins of BMSCs and BMSC-derived exosomes analyzed by Western immunoblotting (*n* = 3). (d) Morphology of MSC-derived exosomes under transmission electron microscopy. Scale bar: 200 nm. (e) The expression level of miR-132 determined by Q-PCR (*n* = 3). ^∗∗∗^*P* < 0.001. NC: negative control.

MSCs were isolated from the bone marrow of C57BL/6 mice as described previously. MSCs were typically spindle-shaped and adherent to the plastic dishes (Figure 1(a)). Flow cytometry was used to identify the surface antigens of MSCs. Results showed that MSCs were negative for CD31, but positive for Sca-1, CD44, and CD105 (Figure 1(b)). The exosomes secreted from BMSCs were isolated as described in Materials and Methods and subjected to biochemical and biophysical analyses. Biochemical analysis of isolated exosomes showed the presence of the exosome proteins CD63 and CD9 (Figure 1(c)), while no expression of CD63 and CD9 was detected in BMSCs. Electron microscopy analysis of exosomes exhibited typical cup-shaped morphology and confirmed the size range of less than 150 nm (Figure 1(d)). Furthermore, a significant miR-132 overexpression was detected by qRT-PCR in electroporated MSCs with miR-132 (Figure 1(e)).

### 3.2. miR-132 Exosomes Are Efficiently Taken Up by HUVECs

**Figure 2:**
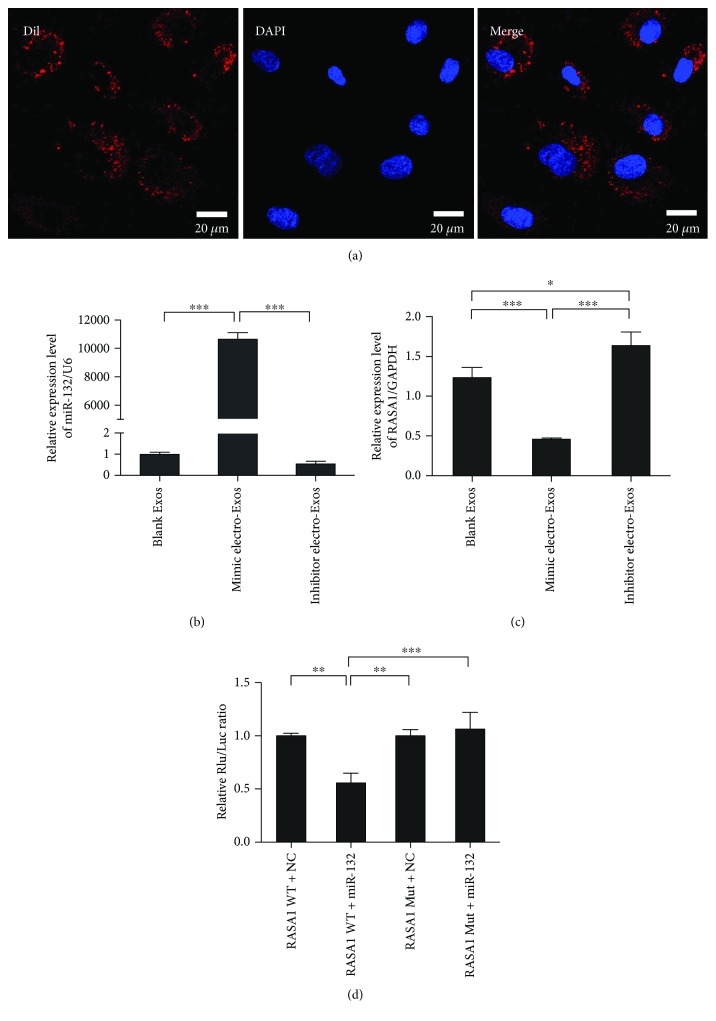
Internalization of miR-132-electroporated exosomes and detection of target gene RASA1. (a) Confocal images of DiI-labelled exosomes taken up by HUVECs. Scale bar: 20 *μ*m. (b, c) HUVECs were incubated with miR-132 mimics or inhibitor-electroporated exosomes for 2 h. The relative expression level of miR-132 and its target gene RASA1 was detected by RT-PCR (*n* = 3). (d) 293T was cotransfected with miR-132 mimics or NC and firefly luciferase reporter plasmid containing wild-type or mutant-type 3′UTR of RASA1. After incubation for 48 h, the firefly luciferase activity of each sample was detected and normalized to the Renilla luciferase activity (*n* = 3). The data represent the mean ± SEM of triplicates. ^∗^*P* < 0.05, ^∗∗^*P* < 0.01, ^∗∗∗^*P* < 0.001.

Exosomes were labelled with CM-DiI dye and incubated with HUVECs *in vitro*. Using an inverted fluorescence microscope, we provided qualitative evidence that HUVECs take up DiI-labelled exosomes derived from BMSCs (Figure 2(a)). Next, we performed qPCR to evaluate the expression of miR-132 in HUVECs. We observed a significant increase of miR-132 expression in HUVECs taking up miR-132 mimic electro-Exos, when compared with both blank HUVECs and HUVECs taking up miR-132 inhibitor electro-Exos (Figure 2(b)). It is worthy of note that the expression of RASA1, a target gene of miR-132, was significantly downregulated (Figure 2(c)). Luciferase reporter assay was used to further confirm the targeting relationship between miR-132 and RASA1. Results showed that miR-132 significantly decreased the relative luciferase reporter activity of the wild-type RASA1 3′-UTR, whereas that of the mutant RASA1 3′-UTR did not change significantly, which suggests that miR-132 could directly bind to the 3′-UTR of RASA1 (Figure 2(d)).

### 3.3. miR-132-Electroporated Exosomes Promote Angiogenesis *In Vitro*

**Figure 3:**
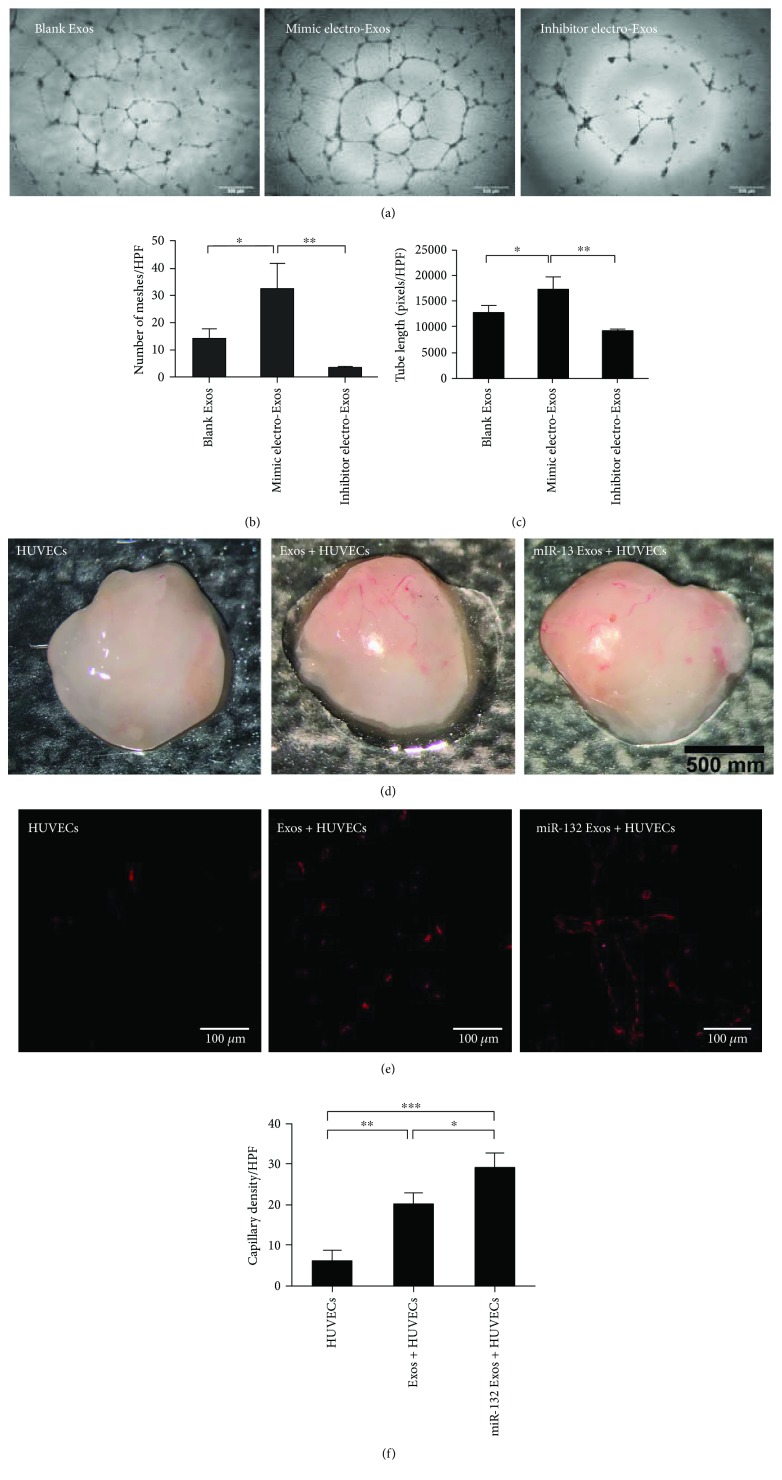
miR-132-electroporated exosomes promoted angiogenesis *in vitro* and *in vivo*. (a) Tube formation assay on Matrigel was assessed 6 h after seeding HUVECs pretreated with blank, miR-132 mimic-electroporated or miR-132 inhibitor-electroporated exosomes. Scale bar: 500 *μ*m. (b, c) Quantitative assessment of the total number of meshes and tube length (*n* = 3). ^∗^*P* < 0.05, ^∗∗^*P* < 0.01. (d) Gross look of Matrigel plugs. (e, f) Immunofluorescence staining of vessels in the sections of Matrigel plugs and quantitative assessment of capillaries per high-power field in each group (*n* = 3). ^∗^*P* < 0.05, ^∗∗∗^*P* < 0.001.

We investigated whether miR-132 electroexosomes could enhance the angiogenic behavior of endothelial cells *in vitro*. The tube length and the number of meshes were increased in HUVECs treated with miR-132 mimic electroexosomes for 12 h, compared to those treated with blank exosomes and miR-132 inhibitor electroexosomes (Figures 3(a)–3(c)). As evidenced by tube formation assay, our study suggests that overexpressed miR-132 could enhance the proangiogenic effects of exosome on endothelial cells.

### 3.4. miR-132-Electroporated Exosomes Promote Angiogenesis *In Vivo*

Finally, we utilized Matrigel plug to examine *in vivo* angiogenic behavior. Matrigel containing HUVECs, HUVECs treated with blank exosome, or HUVECs treated with miR-132 mimic electroexosome was injected subcutaneously into SCID male mice (*n* = 6) in the inguinal regions, respectively. After 14 days, Matrigel was excised and photographed to assess the presence of blood vessels. The Matrigel plugs containing miR-132 exosome exhibited bright red color indicating blood-perfused vessels, whereas blank exosome-containing plugs presented light yellowish color that is correlated with the limited formation of new vessels (Figure 3(d)). In addition, the immunofluorescence staining showed that the number of vessels in the plugs containing miR-132 exosomes (29.33 ± 2.86/HPF) was also significantly higher than that in the negative control (6.33 ± 2.05/HPF) and those containing blank exosomes (20.33 ± 2.05/HPF) (Figures 3(e) and 3(f)).

### 3.5. miR-132-Electroporated Exosomes Preserve Cardiac Function and Promote Angiogenesis in a Mouse MI Model

**Figure 4:**
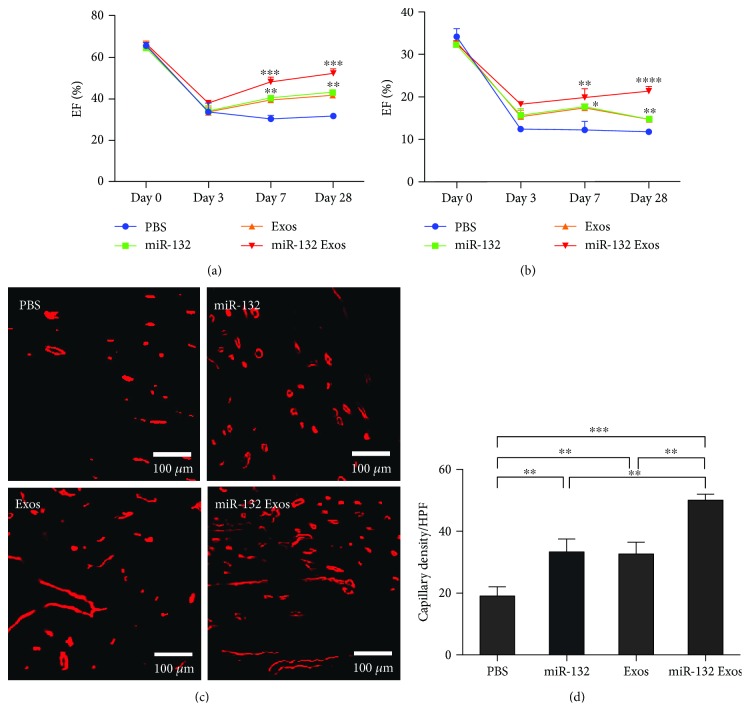
miR-132-electroporated exosomes preserve cardiac function and promoted angiogenesis in MI model. (a, b) Quantitative assessment of LVEF and FS value in each group after MI (*n* = 3). ^∗^*P* < 0.05, ^∗∗^*P* < 0.01, ^∗∗∗^*P* < 0.001, ^∗∗∗∗^*P* < 0.0001. (c, d) Immunofluorescence staining of vessels in the sections of heart tissue and quantitative assessment of capillaries per high-power field in each group. Scale bar: 500 *μ*m (*n* = 3). ^∗∗^*P* < 0.01, ^∗∗∗^*P* < 0.001.

We assessed the *in vivo* therapeutic effects of miR-132 exosomes on a mouse MI model. Preinterventional left ventricular ejection fraction (LVEF) and fractional shortening (FS) values were similar in all groups (data not shown). Significant decreases in LVEF and FS in saline-treated mice were observed on day 7 and day 28 after MI. Compared with saline-treated mice, the miR-132 and normal exosome group partially rescued MI-induced decrease of LVEF and FS, while the miR-132-exosome group significantly increased LVEF (day 7: 30.18 ± 0.94 versus 48.04 ± 1.27, *P* < 0.001, and day 28: 31.56 ± 0.83 versus 51.97 ± 1.32, *P* < 0.001, resp.) and FS (day 7: 12.21 ± 1.16 versus 19.87 ± 1.17, *P* < 0.01, and day 28: 11.80 ± 0.25 versus 21.33 ± 0.64, *P* < 0.0001, resp.) compared with saline-treated mice on day 7 and day 28 after MI (Figures 4(a) and 4(b)). Hearts were excised on day 28 after MI. Capillary density of cardiac tissue was further examined by immunohistochemical stain. Compared with the saline-treated group (19 ± 2.45/HPF), both the miR-132 (33.33 ± 3.40/HPF) and normal exosome groups (32.67 ± 3.09/HPF) had a higher density of vessels. More importantly, the capillary density of the infarct area was significantly increased in the miR-132 exosome group (50 ± 1.63/HPF), (Figures 4(c) and 4(d)). These data demonstrate that miR-132-electroporated exosomes could effectively preserve cardiac function and promote angiogenesis in a mouse MI model.

## 4. Discussion

In this paper, we demonstrated that exosomes loaded with miR-132, as a vehicle for miRNA carriage and transfer, significantly increased tube formation *in vitro* and neoangiogenesis in Matrigel plug and myocardial infarction. Mechanistically, miR-132 promotes angiogenesis by downregulating the expression level of its target gene RASA1 in HUVECs. These findings greatly extend our current understanding of exosomes on angiogenesis and indicate that exosomes give an inspiring hope as vehicles of therapeutic molecules for the treatment of ischemic diseases.

Ischemic heart disease (IHD) is the leading cause of morbidity and mortality worldwide owing to aging, obesity, diabetes, and other comorbid diseases [23]. One potent therapeutic approach for IHD is to induce revascularization, therefore, increase oxygen supply, inhibit cardiomyocyte apoptosis, and reduce myocardial fibrosis. MicroRNAs are small noncoding RNAs that act as negative regulators of protein-coding genes. It has been well established that microRNAs promote both physiological and pathological angiogenesis [4, 24]. A large number of therapeutic strategies based on microRNAs have been carried out on the treatment of myocardial infarction and other ischemic diseases [2, 20, 22].

The intercellular communication occurs directly (between adjacent cells, via gap junctions) or indirectly (at long distances, via soluble factors and extracellular vesicles, including exosomes). These vesicles that act as the vehicles of proteins, RNA, and other molecular constituents modulate the intercellular communication. Previous studies reported that changing the miRNA expression in exosomes derived from MSCs could protect ischemia-reperfusion injury and promote angiogenesis in acute MI [10, 25]. All of these findings indicated that exosomes, as natural therapeutic delivery vehicles, play an important role in angiogenesis [10, 26]. In addition, exosomes can be easily stored at −20°C for at least 6 months without loss of biological activity [27]. Exosomes may be easier to manufacture and standardize in terms of dosage and biological activity.

According to previous researches, we selected miR-132 for gain and loss of function in HUVECs pretreated with electroporated exosomes, to investigate the role of exosome-transferred proangiomiRs in angiogenesis. Exogenous miR-132 mimics and inhibitors were successfully electroporated into MSC-derived exosomes, and it was shown that loaded exosomes can be taken up by HUVECs. The loaded exosomes effectively delivered miR-132 mimics into HUVECs, causing increase of miR-132, and functionally promoted tube formation and neoangiogenesis in Matrigel plug and myocardial infarction. On the contrary, inhibiting the expression of miR-132 in exosomes derived from MSCs resulted in reduced angiogenesis. These findings indicate that the extracellular miR-132 was loaded into exosomes, transferred into endothelial cells, and played a critical role in angiogenesis.

Furthermore, to investigate the molecular mechanisms by which miR-132 might promote angiogenesis, we focus on its target gene RASA1. RASA1 has been reported to be an evolutionary conserved target of miR-132 [5]. Previous studies have demonstrated that RASA1 acts as a crucial negative regulator of vascular sprouting and vessel branching. Furthermore, other researches have revealed that RASA1 regulates endothelial cell behavior during angiogenesis in HUVECs by inactivating the Ras-mitogen-activated protein kinase (MAPK) signaling pathway [5]. Our results showed that increasing the expression of miR-132 led to a statistically significant decrease of RASA1 level in HUVECs. This observation is in agreement with previous data [4]. In order to confirm the interaction between miR-132 and RASA1, we performed dual-luciferase reporter assay which demonstrated that RASA1 is a real target of miR-132 in HUVECs.

In conclusion, we identified that miR-132-electroporated exosomes promoted angiogenesis *in vitro* and *in vivo*. MSC-derived exosomes could be considered as a potential candidate for therapeutic angiogenesis especially for ischemic diseases. Exosomes derived from MSCs have theoretical advantages as a medicinal product, and, in the future, exosomes may gain preference over whole cell-based therapy in the discipline of regenerative medicine.

## Data Availability

The data used to support the findings of this study are available from the corresponding author upon request.

## Abbreviations

MSC: Mesenchymal stem cells
MI: Myocardial infarction
3′-UTR: 3′-untranslated region
VEGF-A: Vascular endothelial growth factor A
RASA1: p120RasGap
MeCP2: Methyl-CpG-binding protein 2
MAPK: Ras-mitogen-activated protein kinases
DMEM: Dulbecco's modified eagle medium
HUVECs: Human umbilical venous endothelial cells
TEM: Transmission electron microscope
PFA: Paraformaldehyde
LAD: Left anterior descending artery
DAPI: 4,6-Diamidino-2-phenylindole
BSA: Bovine serum albumin
WT: Wild-type
PVDF: Polyvinylidene difluoride
LVEF: Left ventricular ejection fraction
FS: Fractional shortening
IHD: Ischemic heart disease.
